# Genomic Analysis of *Pseudomonas putida* Phage tf with Localized Single-Strand DNA Interruptions

**DOI:** 10.1371/journal.pone.0051163

**Published:** 2012-12-07

**Authors:** Anatoly S. Glukhov, Antonina I. Krutilina, Michael G. Shlyapnikov, Konstantin Severinov, Daria Lavysh, Vladimir V. Kochetkov, John W. McGrath, Colin de Leeuwe, Olga V. Shaburova, Victor N. Krylov, Natalia V. Akulenko, Leonid A. Kulakov

**Affiliations:** 1 Institute of Protein Research, Russian Academy of Sciences, Pushchino, Russia; 2 Skryabin Institute of Biochemistry and Physiology of Microorganisms, Russian Academy of Sciences, Pushchino, Russia; 3 Institutes of Molecular Genetics and Gene Biology, Russian Academy of Sciences, Moscow, Russia; 4 Waksman Institute, Rutgers, The State University of New Jersey, Piscataway, New Jersey, United States of America; 5 School of Biological Sciences, The Queen’s University of Belfast, Belfast, Northern Ireland, United Kingdom; 6 Department of Microbiology, Laboratory for Genetics of Bacteriophages, I.I. Mechnikov Research Institute for Vaccines and Sera, RAMS, Moscow, Russia; University of Massachusetts Medical School, United States of America

## Abstract

The complete sequence of the 46,267 bp genome of the lytic bacteriophage tf specific to *Pseudomonas putida* PpG1 has been determined. The phage genome has two sets of convergently transcribed genes and 186 bp long direct terminal repeats. The overall genomic architecture of the tf phage is similar to that of the previously described *Pseudomonas aeruginosa* phages PaP3, LUZ24 and phiMR299-2, and 39 out of the 72 products of predicted tf open reading frames have orthologs in these phages. Accordingly, tf was classified as belonging to the LUZ24-like bacteriophage group. However, taking into account very low homology levels between tf DNA and that of the other phages, tf should be considered as an evolutionary divergent member of the group. Two distinguishing features not reported for other members of the group were found in the tf genome. Firstly, a unique end structure – a blunt right end and a 4-nucleotide 3′-protruding left end – was observed. Secondly, 14 single-chain interruptions (nicks) were found in the top strand of the tf DNA. All nicks were mapped within a consensus sequence 5′-TACT/RTGMC-3′. Two nicks were analyzed in detail and were shown to be present in more than 90% of the phage population. Although localized nicks were previously found only in the DNA of T5-like and phiKMV-like phages, it seems increasingly likely that this enigmatic structural feature is common to various other bacteriophages.

## Introduction

The genus *Pseudomonas* includes several medically and environmentally important species and bacteriophages have been isolated for most of them. Bacteriophages of *P. aeruginosa* attracted most of the attention as they are widespread and considered to be of potential therapeutic importance in fighting antibiotic resistant strains. Various groups of lytic phages specific to *P. aeruginosa* were characterized and amongst these were the giant phiKZ-like Myoviridae phages possessing some of the largest viral genomes known [1]. The family of Podoviridae is also well represented amongst known *P. aeruginosa* phages. The phiKMV-like viruses of *P. aeruginosa* belonging to this family were thoroughly studied; they are ubiquitous in nature and infect a wide range of host strains [2], [3]. Bacteriophages LUZ24 [4] and PaP3 [5] represent another interesting group of Podoviridae infecting *P. aeruginosa*. Though isolated from geographically distant regions, these phages share a high level of DNA identity (71%). Their ∼46 kbp genomes are transcribed bidirectionally [5], [4]. It is notable that while LUZ24 appears to be a lytic phage [4]; PaP3 was reported to be able to integrate into the host genome similarly to temperate bacteriophages [5]. A sequence of the bacteriophage phiMR299-2 which has 92.8% identity with that of PaP3 has been published recently [6].

Bacteriophages of *P. putida* so far have been studied to a much lesser extent. A T7-like lytic phage gh-1 of *P. putida,* which was isolated by Lee and Boezi [7], has been characterized by Kovalyova and Kropinski [8]. More recently, Cornelissen et al. [9] reported a sequence of the phi15 phage. The genomes of both phages consist of dsDNA and are about 55% identical to each other. In both phage genomes, the genes are laid out in a manner characteristic to other T7-like viruses [8], [9].

The lytic bacteriophage tf belonging to the Podoviridae family and specific to *P. putida* PpG1 was previously described by Kulakov et al. [10]. The phage genome comprised of a dsDNA with several localized nicks detected [11]. Here, we characterize the genomic organization of this phage. The tf genome has a significant similarity to LUZ24-like phages. However, its genome has a number of localized single-strand interruptions as well as unique ends, features not reported for either LUZ24 or PaP3 genomes.

Localized single-chain interruptions (sci) were previously considered to be unique for T5 and its relative BF23 [12], [13], [14]. Biological significance of the site-specific nicking of the T5 DNA has not been revealed despite extensive studies of this phage [14]. Investigation of *P. aeruginosa* bacteriophages phikF77, phiKMV, LUZ19 and LKD16 revealed that they also have canonical nicks located in the non-coding DNA strand [15]. These nicks were shown to be present with various frequencies within the phage phikF77 population. A consensus sequence in the nicks (5′-CGACxxxxxCCTA/CTCCGG-3′) was found in all phages of the group [15]. As in the case of T5, the biological function of localized nicking in phiKMV-like phages remains unknown. Our current findings suggest that site-specific nicking is a common feature present in genomes of bacteriophages belonging to various groups specific to at least three bacterial species and thus call for further studies.

## Methods

### Bacteriophage Growth and Purification

*Pseudomonas putida* PpG1 strain was used as a host for the bacteriophage and cultivations were conducted in a rich 2YT medium [16]. The bacteriophage preparations were concentrated with polyethylene glycol (PEG6000) and phage particles purified using standard Cesium Chloride gradient technique [17].

### Phage Analysis and DNA Techniques

Bacteriophage DNA was isolated from CsCl gradient purified viral particles as described by Sambrook et al. [17]. Standard methods of DNA manipulation were used throughout this work [17]. All restriction endonucleases as well as T4 DNA ligase were obtained from Thermo Scientific UK. For visualization and analysis of localized single-strand interruptions bacteriophage DNA preparations were denatured by alkali (treatment with 0.2 M NaOH for 5 min at room temperature) prior to separation in 0.9% agarose gel. SDS-PAGE analysis of the phage’s structural proteins was conducted according to a standard protocol [17] using 20 µl of CsCl gradient purified phage preparation (5×10^11^ pfu/ml).

### Electron Microscopy

For EM analysis of the DNA molecules a Jeol-100 microscope was employed. Samples were prepared using bacteriophages purified by CsCl gradient centrifugation (10^9^ pfu/ml) and resuspended in a buffer (50 mM Tris-HCl, pH 8.5, 10 mM EDTA) containing formamide. The formamide concentration, temperature and time of denaturing were varied to achieve conditions at which single-strand interruptions in DNA were visualized and at the same time no melting of AT rich regions occurred. DNA denaturation was conducted for 12 min at 37°C in 85% formamide. Samples were further prepared as described by Davis et al. [18].

### Genome Sequencing and Analysis

The genome sequencing of phage tf was performed using a combination of shot-gun clone library sequencing and Genome walking. Initially libraries were constructed by cloning blunt end fragments generated by restriction endonucleases RsaI, HpaI, EcoRV and SmaI into pJET1.2 vector (Fermentas). Cloning was performed using CloneJET™ PCR Cloning Kit (Fermentas) according to the manufacturers instructions. Based on the sequencing information obtained, primers for the Genome walking were further designed. Both strands of phage DNA were sequenced.

Sequences were assembled with CodonCode Aligner (Codon Code Corp., USA). Searches for nucleotide and amino acid sequence similarities were carried out using the BLAST program [19] in GenBank (blastn and blastx algorithms). Alignments of the sequences were done using the CLUSTALW algorithm [20]. ORFs of the tf genome were predicted using the GeneMark server (http://exon.gatech.edu/gmhmm2_prok.cgi) and ORF finder (http://www.ncbi.nlm.nih.gov/gorf/orfig.cgi). The BLASTP and PSI-BLAST algorithms were used to detect homologs of tf genes in the nonredundant GenBank protein database. RNA genes were searched using the tRNAscan-SE server (http://lowelab.ucsc.edu/tRNAscan-SE/).

To identify σ^70^-dependent putative promoters we used a positional weight matrix (pattern), describing the –10 and –35 elements of *E. coli* σ^70^ promoters. The matrix was constructed using the DPInteract database collection of known *E. coli* σ^70^ promoters [21]. The promoter pattern was used to search the tf genome with the Genome Explorer program [22]. The following search parameters were employed: (*i*) the spacer length between the –10 and the –35 promoter elements was allowed to vary from 16 to 19 bp; (*ii*) the search was bidirectional. The search cutoff was set at a conservative site score of no less than 5.31. The genome was scanned for conserved intergenic motifs using the MEME/MAST algorithm (http://meme.sdsc.edu/meme/cgi-bin/meme.cgi). The bacteriophage genome sequence is deposited to the European Nucleotide Archive under Accession Number HE611333.

### Mapping of Localized Nicks and Quantification of their Frequencies

Mapping of the nicks was performed by sequencing using phage specific primers and native tf DNA as a template. Nicks were identified by drops of signals in the corresponding sites. Quantification of the nicks’ frequencies in phage DNA populations was determined for two nicks (sci-2 and sci-14). To achieve that primer extension reaction was employed. *fmol*® DNA Sequencing System obtained from Promega and specific 5′-[^32^P] labeled primers: tf_sci-2∶5′-TAGCCATCAGTCACCACCA-3′ and tf_sci-14∶5′-GGGTAACCAGCCAGAGTGT-3′ were used. To quantify the efficiency of the nicking primer extension reaction were also conducted using tf DNA preparations hydrolyzed with *Hae*III restriction endonuclease and the corresponding [^32^P] labeled primer. Products of these reactions were analyzed using 6% polyacrylamide gel electrophoresis (PAGE) in denaturing conditions and gels were autographed with the Cyclone Storage Phosphor System (Packard Instruments Co). Estimation of the percentages of DNA molecules with localized single-chain interruptions was conducted using OptiQuant software.

### Sequencing of the Phage DNA Ends

To determine 5′-end sequences of the tf genome primer extension reactions were conducted with 5′-[^32^P] labeled primers: 5′-tf_r: 5′-CCCCGGATGATAGCATG-3′ and 3′-tf_l: 5′-CCCTGTGTATTCATCCAGTG-3′. *fmol*® DNA Sequencing System obtained from Promega was employed and the reactions’ products were analyzed using 6% PAGE in denaturing conditions and gels were autographed with the Cyclone Storage Phosphor System (Packard Instruments Co).

To determine 3′-end sequences of the phage genome denatured tf DNA preparations were polyadenylated with Terminal deoxynucleotidyl transferase (TdT) obtained from Promega using conditions recommended by manufacturer. Products obtained were used as templates in PCR reactions with one Poly dT primer and one primer specific to the corresponding end: tf_l: 5′-TTGAATTCGATACCACAGTGTACCCA-3′ (left end) and tf_r: 5′-TTTGTCGACCCTAAAGTTCATTTCTTTCT-3′ (right end). PCR products obtained were analyzed by sequencing.

## Results

### Genome Sequence Analysis

A Genome walking approach using native phage DNA preparations was employed for sequencing of the phage in order to detect all localized nicks (single-chain interruptions) present. The nucleotide sequence of dsDNA of the tf phage was determined to be 46,267 bp. The genome contains 186 bp long direct terminal repeats. A G+C content of the tf genome is 53.2%, which is markedly different from the 61.5% value reported for *P. putida* [23].

**Figure 1:**
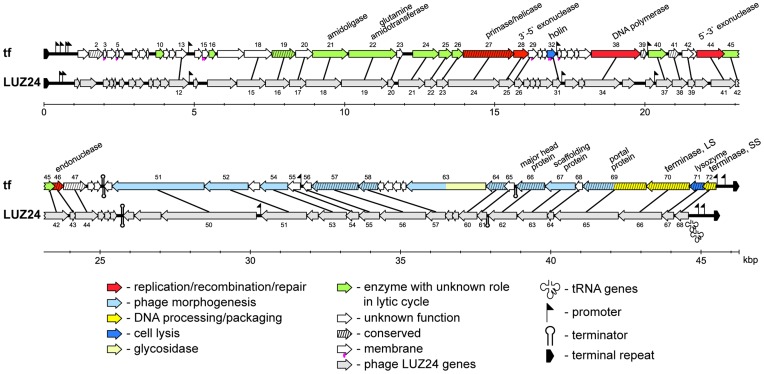
Genome organization of tf and its comparison with LUZ24 bacteriophage. Predicted genes are designated with arrows indicating their directions of transcription. Color and symbol codes adopted are shown at the bottom of the figure. The similarities between corresponding amino acid sequences of tf and LUZ24 are indicated by connecting lines.

72 ORFs were identified in the tf genome (Fig. 1 and Table S1). 61 of predicted tf ORFs initiated from ATG start codons, seven – from GTG, and four – from TTG. Translation was terminated with TAA (38 ORFs), TGA (32) and TAG (2) stop codons. Overlapping ORFs are common for phages; several overlapping genes are present in tf. In most cases, a minimal overlap of start and stop codons was observed. Such organization may be explained by translation of mRNA by re-initiation of translation without ribosome dissociation according to scanning model of translation in prokaryotes [24]. The maximum overlap of 137 bp was detected between *tf*.45 and *tf*.46.

The deduced amino acid sequences of more than half of the tf ORFs (39 ORFs) were similar to those of previously reported LUZ24 and PaP3 phages. It is notable that orthologous genes have the same arrangements in this group of phages (Fig. 1). As in LUZ24 and PaP3, the tf genes presumably encoding early functions are located in the left half of the genome and transcribed in one direction, while those encoding late functions are clustered in the right half of the genome and are transcribed in the opposite direction ([4]; Fig. 1). Thus, tf belongs to LUZ24-like phages. However, tf DNA has little overall similarity to LUZ24 and PaP3 DNA (52.5% and 51.5% respectively; compared to 70.1% identity of LUZ24 and PaP3 DNA). Thus, tf is a distant relative of other members of the group.

Analysis of the nucleotide sequence allowed to localize seven putative promoters recognized by host RNA polymerase σ^70^ holoenzyme in the left half of the genome and three convergent promoters in the right half. Each putative promoter contained the –10 and –35 elements matching the consensus sequences separated by spacers of appropriate length. The positions of all putative promoters in the tf genome correspond to those of experimentally identified promoters in LUZ24 [4]. Overall, it appears that the tf genome is organized into two converging transcription units. This is also the case for PaP3 and LUZ24 [5], [4]. Convergently transcribed tf genes are separated by a predicted rho-independent bidirectional terminator (position 25092-25133; Table S1).

49 ORFs were localized in the left transcription unit. Functions were predicted for eight genes. In another twelve known amino acid sequence domains/motifs were identified (Table S1). There is a block of genes with the products known to participate in replication and recombination of DNA; these are genes coding for DNA polymerase (gp38), 5′-end (gp44) and 3′-end endonucleases (gp28), primase/helicase (gp27), endonuclease related to bacteriophage T7 GP3 Family (gp46). A gene coding DNA binding protein gp40 belongs to the same cluster. There are also genes of possible bacterial origins such as amidoligase and glutamine amidotransferase.

The right transcription unit consists of 23 genes (Fig. 1; Table S1). The order of the genes in this region was typical for packaging and DNA processing genes followed by virion genes coding for portal, head and finally putative tail and tail fiber proteins. ESI-MS/MS analysis of LUZ24 virions identified 9 proteins as components of the phage particle [4]. 7 tf proteins were found to be highly homologous to LUZ24 virion proteins. No homolog of LUZ24 virion protein gp49 is encoded by the tf genome; LUZ24 virion protein gp52 is likely substituted by a non-homologous protein of the same length encoded by the *tf*.53 gene. LUZ24 genes 49 and 52 are located at the distal part of late gene cluster where tail and tail fiber genes are usually situated. Considering that LUZ24 and tf are specific to different species of *Pseudomonas* it could be suggested that genes *tf*.53 (tf) and orf49 and orf52 (LUZ24) are responsible for the host range of these phages.

Out of the total of 72 tf ORFs identified, 32 were unique for the tf genome and 23 were conserved in other phages. Most of the unique genes are located in the left early transcription region. This pattern was reported for LUZ24 and PaP3 phages. All but one (*tf*.63) unique tf genes encode proteins lacking homologues in protein databases. Several predicted proteins could represent membrane proteins (gp29, gp54) and secreted proteins (gp32, gp59 and gp62).

The *tf*.63 gene is located in the right transcription region and codes for a putative hydrolase belonging to the SGNH protein family (Table S1) which includes diverse lipases and esterases. Similar hydrolases encoded by some *Bacillus* phages were suggested to improve phage adsorption by degrading capsule polymers of γ-linked glutamate and poly-γ-glutamate [25]. There were reports that exopolysaccharides (EPS) of *P. putida* play an important role in stabilization of biofilms [26], [27]. It was demonstrated recently that EPS degrading activity is present in the tail-spike protein of the *P. putida* phage phi15 [9]. This activity was also shown to be instrumental for the degradation of biofilms formed by phi15 host [9]. We suggested that gp63 may be part of the tf virion. SDS-PAGE analysis of proteins of tf virions (Fig. S1) demonstrated a protein with a molecular weight corresponding to that of the *tf*.63 product, suggesting that gp63 is a unique tf virion protein with a functionality which may be similar to that phi15 particle-associated EPS depolymerase.

It is usual for DNA polymerases to combine polymerase and proofreading 3′-exonuclease activities in the same polypeptide chain. However, in all phages of the LUZ24 group they are represented by separate genes. Similar organization was reported for the *E. coli* phiEco32 phage [28]. It is notable that in LUZ24 an intron was found in the DNA Pol gene whereas the corresponding genes in both PaP3 and tf are not interrupted.

### Determination of the DNA Ends Structure

**Figure 2:**
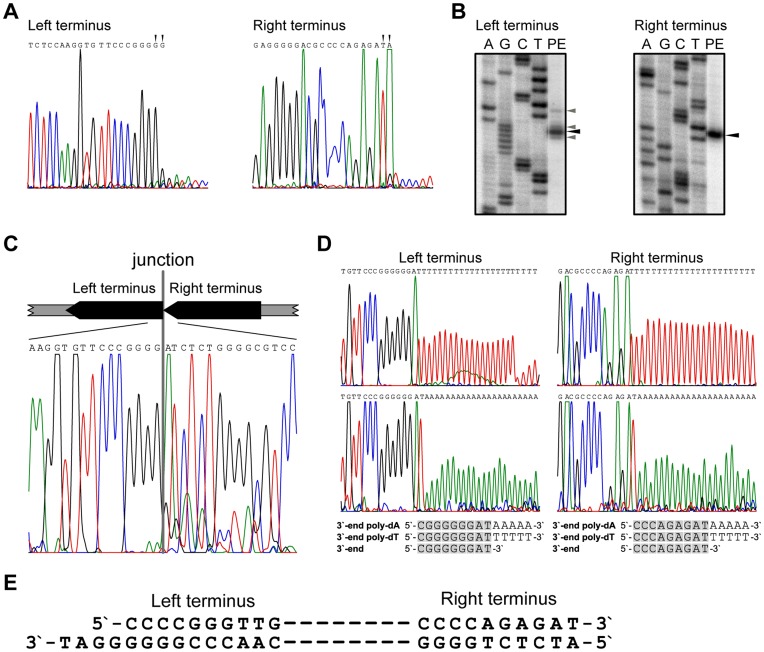
Analysis of the tf DNA ends. (A) Automated sequencing of the bacteriophage tf DNA at termini regions. Alternative end-nucleotides are indicated. (B) Results of the primer extension analysis of the 5′-ends in the tf DNA. Nucleotide positions corresponding to the stoppages at the phage DNA ends are indicated with arrows; black arrow for the position corresponding to the major band and gray arrows – position corresponding to minor bands at the left terminus only. (C) Analysis of the termini junction in the circularized tf DNA. Bacteriophage DNA was treated with T4 DNA polymerase and circularized using T4 DNA ligase. The junction region was analyzed by automated sequencing of the corresponding PCR product as described in the text. The junction identified is shown on the chromatogram by a vertical line and the schematic of the ligated genome ends is shown above the chromatogram. (D) Analysis of the 3′-ends in the tf DNA. Denatured tf DNA was treated with TdT enzyme in separate experiments in the presence of either dATP or dTTP and PCR products were obtained using resulting preparations and one phage specific and one poly-dT (poly-dA) specific primer. Chromatograms for all types of experiments are presented with the corresponding sequences shown at the bottom of the figure. Terminal sequences of the phage genome are shown by shading. (E) Structure of the tf DNA ends’ sequences.

Preliminary data concerning 5′-end structures of phage DNA was obtained in the course of run-off sequencing of the corresponding chromosomal regions (Fig. 2A). It is known that Taq DNA polymerase often adds an extra non-templated nucleotide to the 3′-end of the synthesized DNA [29]. Taking this into account, the 5′-sequence at the left end of tf phage chromosome could be either 5′-CCCCCG-3′ or 5′-CCCCG-3′, and at the right end –5′-TATCTC-3′ or 5′-ATCTC-3′ (putative 5′-end nucleotides are underlined). Similar conclusions were reached when the products of the corresponding primer extension reactions were analyzed (Fig. 2B). It is worth noting that primer extension results indicated a possible heterogeneity of the left 5′-end of phage DNA and homogeneity at their right ends where more than 99% of the molecules had identical sequences (Fig. 2B). To determine the precise structures of 5′-ends, the preparations of native tf DNA were treated with T4 DNA polymerase in order to produce blunt ended DNA molecules (by filling in possible 5′-protruding ends and/or removal of 3′-protruding ends). The subsequent treatment with T4 DNA ligase was employed to circularize blunted tf DNA molecules. Resulting preparations were used for PCR amplification with phage specific primers annealing outside Direct Terminal Repeats. Sequencing analysis of PCR products obtained allowed to localize the junction points between the left and the right ends of phage chromosome (Fig. 2C) and determine the precise sequences of genome ends as 5′-CCCCGG-3′ (left end) and 5′-ATCTC-3′ (right end). Possible heterogeneity at the left terminus (Fig. 2B) was estimated to affect no more than 5% of phage population (estimated using OptiQuant software; see Material and Methods).

In the recent review of phage DNA end organization it was noted that phages with short exact direct repeat ends (similar to tf) “are thought to have blunt ends” [30]. However, we considered a possibility that tf DNA had either non-complementary protruding ends or one blunt and one protruding end. To investigate this, the 3′-end structures at the tf DNA ends were analyzed.

The extension reaction of the DNA 3′-ends with terminal deoxynucleotidyl transferase was employed. Both poly-dT and poly-dA extended molecules were synthesized in two different experiments in order to take into account possibilities of the 3′-end nucleotide being either “A” or “T”. The sequencing analysis and comparison of PCR fragments obtained with one phage-specific and one homopolymer complementary primer was conducted which allowed to determine positions of the junctions between corresponding homopolymers and the last 3′-end nucleotide of the phage chromosome (Fig. 2D).The structure of the tf DNA ends was determined and is presented in Fig. 2E. Our results indicate that the tf genome has a blunt right end and a 4-nucleotide 3′-protruding left end. To the best of our knowledge no similar end structure was previously shown to be present in a phage chromosome.

### Identification and Analysis of Localized Nicks in the tf Genome

**Figure 3:**
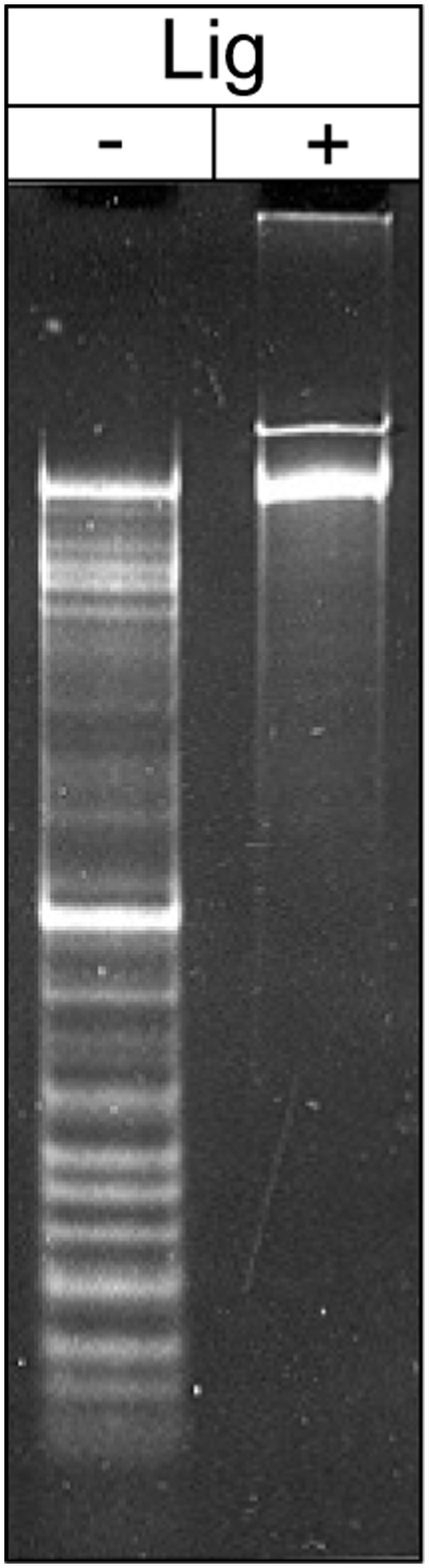
Identification and analysis of the localized nicks in tf DNA molecules. DNA preparation of bacteriophage tf was denatured in 0.1 M NaOH and subjected to electrophoresis in 0.9% agarose gel; in a control experiment a DNA from the same preparation was treated with T4 DNA ligase prior to denaturing in 0.1 M NaOH and electrophoresis.

The analysis of tf sequencing data revealed significant drops of signals in a number of places. These were observed only when sequencing was performed on the native DNA preparations using primers complementary to the top strand (Fig. S2). This observation could be explained by stoppages of DNA polymerase during read-through of sites with single-chain interruptions present in identical positions in the majority of DNA molecules being sequenced. To prove this we analyzed denatured phage DNA by electrophoresis. As shown in Fig. 3, denaturation of the tf DNA resulted in the appearance of a number of bands, which were absent when DNA pre-treated with T4 DNA ligase was denatured. This demonstrated that localized nicks with adjacent 3′-OH and 5′-P groups are present in phage DNA and are likely to be located in one strand only.

Sequence analysis of putative nicked regions identified the consensus sequence as 5′-TACTRTGMC-3′. We analyzed the complete tf nucleotide sequence and found eleven 5′-TACTGTGAC-3′, two 5′-TACTGTGCC-3′ and one 5′-TACTATGAC-3′ sites (Table 1). All such sites were present only in the top and not in the bottom strand of tf DNA. Additional primers were designed where needed and sequencing with these primers confirmed that nicks are present in all fourteen sites (Fig. S2).

**Table 1:** Putative nickase recognition sites in the genomes of LUZ24-like phages.

Site/position in the phage genome (%)*											
5′-TACTGTGAC-3′				5′-TACTATGAC-3′				5′-TACTGTGCC-3′			
tf	LUZ	PaP3	MR	tf	LUZ	PaP3	MR	tf	LUZ	PaP3	MR
				4.5	4.2	2.0	2.0				
7.6					7.3	6.9	5.2				
9.1					11.7	12.0	10.9				
11.4					14.1	19.6	18.5				
	18.5					24.5	23.8	14.3			
18.7					29.1	30.3	29.5				
21.5					45.5	45.5	52.3				
			63.0		55.6	52.8		27.1			
28.8	65.4	63.2									
33.9											
44.9											
53.4											
73.2											
80.2											

* ,nucleotide sequences of the PaP3 (Accession number AY078382) and phiMR299-2 (JN254801) are reverse complemented.

It is notable that 11 out of 14 nicks are associated with a 5′-TACTGTGAC-3′ sequence and the remaining three have a single substitution in that sequence. Based on this observation we decided that all single substitutions from the 5′-TACTGTGAC-3′ sequence needed to be considered as potential recognition sites for phage nickase. Only three additional sequences were found: 5′-CACTGTGAC-3′ (9316–9324), 5′-TATTGTGAC-3′ (25082–25090), and 5′-TACCGTGAC-3′ (13620–13628). Sequence analysis has not detected any decreases in signal at these sites, indicating that a vast majority of the phage genomes are intact at these points (data not presented).

**Figure 4:**
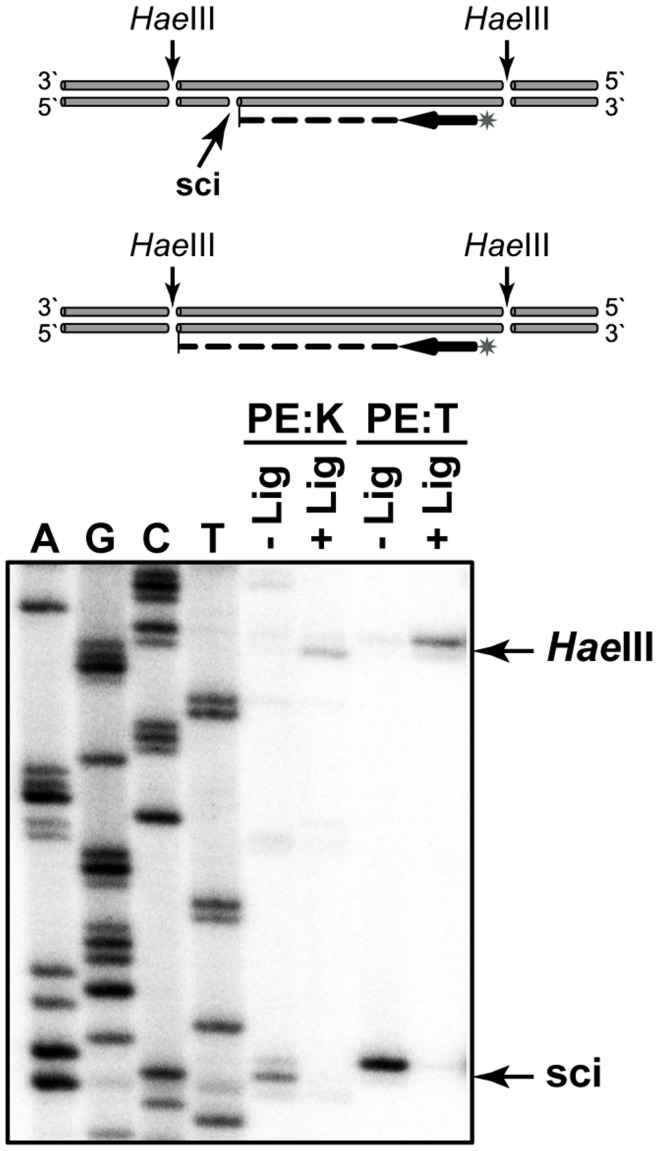
Primer extension analysis of the sci-14. The schematic of the experiment is shown at the top of the Figure: non-ligated (1) and ligated (2) DNA preparations were digested with HaeIII restriction endonuclease and primer extension reaction were performed using the same [^32^P] labeled primer. The results of primer extension with Klenow fragment (PE:K) and Taq DNA polymerases (PE:T) are presented at the bottom part of the Figure. Bands corresponding to the stoppages at the sci-14 and *Hae*III sites are indicated by arrows. Sequencing ladders (A, G, C, T) were generated by Sanger sequencing of the T4 DNA ligase treated tf DNA employing the same [^32^P] labeled primer used in primer extension.

The described sequencing data in principle located nicks in the 5′-TACT/RTGMC-3′ sequence. However, to verify position of the nicks and to quantify the frequency of their occurrence in the phage DNA, primer extension analysis was performed with Klenow fragment and Taq DNA polymerases for sci-2 and sci-14. For both sites, primer extension analysis with appropriate primers was performed using tf DNA preparation digested with HaeIII as a template. Identical data were obtained for both nick sites and data for sci-14 are shown in Fig. 4. The clearest results were obtained with Taq DNA polymerase: a single band corresponding to the nick position and almost total absence of a band corresponding to the HaeIII cleavage point could be seen. Quantification suggested that sci-14 is present in at least 95% of phage DNA molecules. It is known that Taq DNA polymerase usually adds an additional nucleotide (A) at the 3′-end due to the absence of proofreading activity. This addition is clear from the comparison with primer extension results obtained with the Klenow fragment (Fig. 4). Overall, the data confirm that nicks are always introduced into 5′-TACTRTGMC-3′ sequence after the fourth position and, secondly, that they are present in the majority of phage DNA molecules.

**Figure 5:**
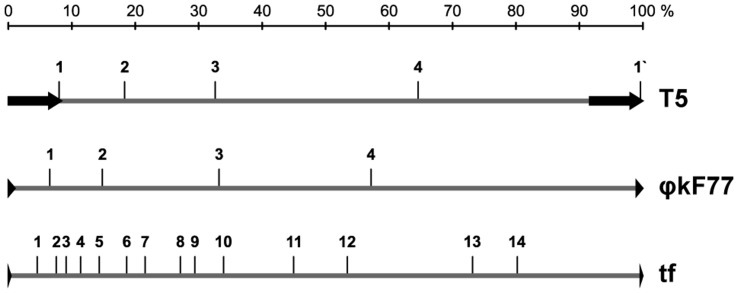
Comparison of localized nick (sci) distribution in genomes of the phages T5, phikF77 and tf. Nicks are shown as long vertical lines. Only “Major” nicks are shown in T5 chromosome. Direct terminal repeats (DTR) found in these phages are indicated with arrows. Locations of all nicks are given as the percentages of the corresponding genome size.

**Figure 6:**
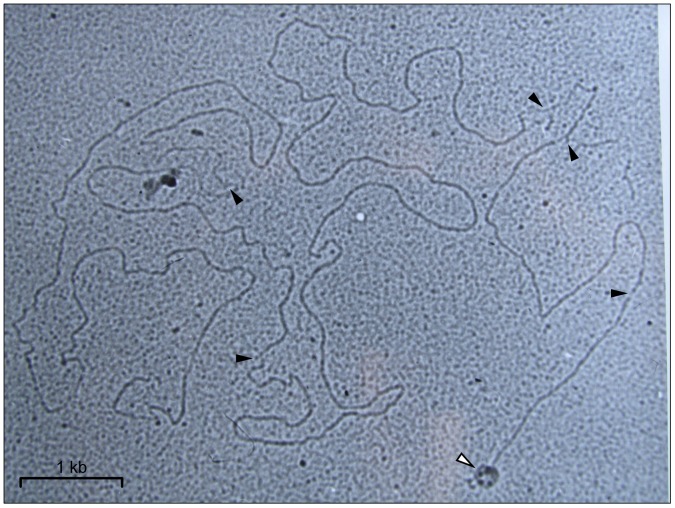
Electron Microscopy of partially denatured tf DNA molecules. Nicks are visualized as partially denatured regions which are indicated by black arrows. Bacteriophage head attached to DNA molecule is indicated by white arrow.

The data presented in Table 1 and Fig. 5 showed uneven distribution of nicks in the phage tf DNA. Eleven out of fourteen nicks were localized in the left half of the molecule. This asymmetry allowed us to investigate the polarity of DNA packaging using electron microscopy of partially denatured DNA. To do that, only DNA molecules attached to phage particles were studied. In all cases analyzed, DNA was attached to phage heads by its left end (Fig. 6). This finding alone does not determine the polarity of DNA packaging or injection during the tf infection of the host cells. However, it is worth noting that when similar experiments were conducted for T5 the left end of the phage DNA was identified as a head-attached one; and this was shown to be the end which enters the cell first during infection [31], [32]. To draw definite conclusions regarding tf, further experiments directly addressing the polarity of its DNA injection are needed.

## Discussion

In this work we characterized a new representative of the LUZ24-like phage group. At present, the group comprises four phages and includes both temperate and lytic phages. Although this is not a widespread phenomenon, homologies between genomes of lytic and temperate phages have been reported previously. There were large regions of homology found between lytic (DT1) and temperate (O1205) phages of *Streptococcus thermophilus* [33] and recently sequenced *P. aeruginosa* lytic phage PA1Ø was found to be 90% homologous to a well characterized phage-transposon D3112 [34]. PaP3 was the first identified representative of the LUZ24 group and it was shown to be able to integrate into host DNA. PaP3 phage attachment site (*att*P) is located in the gene encoding tRNA^Pro^. A 21 bp sequence of this tRNA gene is identical to the tRNA^Lys^ gene in the bacterial attachment site (*att*B). No stable PaP3 lysogens were isolated [5]. In fact, no typical genes needed for establishment and maintenance of lysogeny were identified in any representative of the LUZ24-like phages. Apparently, PaP3 does not form typical lysogens. Indeed, it was shown that PaP3 sequences flanked by host DNA were present in lysogenised strain in quantities that were much higher than equimolar [5], suggesting multiple integration events. The reasons for different developmental strategies used by LUZ24-group phages are not known. Compared to PaP3, LUZ24 carries a single substitution in the 21 bp *att*P site and several substitutions in the flanking regions, which may be enough to prevent integration as has been shown for the much studied lambda phage [35]. There is no tRNA genes present in the tf genome, which may explain its strictly lytic nature.

There are two convergent transcription units in tf and the same type of genome organization was previously shown for PaP3 [5] and LUZ24 [4]. Elucidation of the mechanism(s) of temporary regulation of gene expression in these phages is of considerable interest and must await identification of phage-encoded transcription factors.

Here, we demonstrated that the bacteriophage tf has fourteen single-chain interruptions in its DNA. Canonical nicks were first discovered in T5 and in its close relative bacteriophage BF23 in the 1960s, and this feature was considered to be unique for these phages for a long time [12], [13], [14]. Later, *P. aeruginosa* phiKMV-like phages were also found to contain single-chain interruptions in their DNA [36], [15].

Nicks were found for T5 its relative BF23 and all phiKMV-like phages [14], [15]. Therefore the presence of sci in DNA may be considered as a systematic feature for those two groups. As is shown here, tf belongs to the group which includes three other phages: PaP3, phiMR299-2, and LUZ24. So far, nicks were not reported for either of these phages [4], [5], [6]. Nine copies of the sequence corresponding to the tf consensus are present in both PaP3 and LUZ24 phages and eight such sequences are identified in the phiMR299-2 genome (Table 1). Similar to tf, T5-like and phiKMV-like phages, all these sites are located in one DNA strand only. It is notable that positions of the putative nickase recognition sites are similar in all four phages of the LUZ24 group (Table 1). However, the main putative recognition site found in PaP3, phiMR299-2 and LUZ24 (5′-TACTATGAC-3′; Table 1) differs from that of tf (3′-TACTGTGAC-3′; Table 1). The number of the sites in these phages also differs. Therefore it will be important to investigate the presence of sci in PaP3, phiMR299-2 and LUZ24.

Two types of nicks were reported for the bacteriophage T5; five major nicks were found in DNA with the frequencies of 80% to 90% and a number of minor nicks that appear with much lower frequencies (5%–10%). These frequencies were determined using electron microscopy of the partially denatured DNA molecules [32], [14]. In tf, all 14 nicks are apparently present in the majority of the DNA molecules with a frequency close to 100%. We do not have data which allows the identification of possible minor nicks in tf DNA as these would not be detectable by sequencing. Detection of minor nicks in denatured tf DNA (Fig. 3) or employing electron microscopy of partially denatured molecules also encounters significant difficulties because of the large number of major nicks compared to T5 and phikF77 where such approaches were successful.

Most of tf nicks occur within a 5′-TACTGTGAC-3′ consensus sequence; however, two substitutions (in positions 5 and 8) apparently do not lead to reduction of the frequency of the nicking. As discussed above, we cannot be sure that all possible nicking sites were identified in tf DNA as nicks with low frequencies of occurrence in DNA populations were reported for T5-like phages [32]. However, results of electrophoretic separation of denatured tf DNA (Fig. 3) and sequencing data suggest that all or nearly all nicks were identified. It is possible to hypothesize that co-evolution of nickase’s sequence requirements and cognate sites took place in the case of bacteriophage tf. Two results support this hypothesis. Firstly, there are only seventeen 5′-TACTGTGAC-3′-based sites including any single nucleotide substitution and all of them are located in the same DNA strand. And, secondly, sequences with substitutions in positions 1, 3 and 4 are not recognized by tf nicking system. When compared to bacteriophage T5, these data suggest a different evolutionary pathway of the nicking system. Consensus sequence for four T5 major nicks were deduced as 5′-R/GCGCRGG-3′ (Ksenzenko et al. A/c No AY543070). This seven nucleotides’ sequence occurs five times in a nick-less strand of T5 DNA which suggests that additional sequences should be involved in the site recognition by the T5 nicking system. It could also be suggested that site recognition in T5 can tolerate various substitutions resulting in a large number of so called “minor” nicks found in the phage DNA [32]. As for *P. aeruginosa* phiKMV-like phages their nickase system [15] resembles that of tf described here.

The biological function of the nicks is still not clear even for T5-like phages. A number of hypotheses were suggested and two genes (*sciA* and *sciB*) responsible for the nicking were identified and mapped to the late region [37]. Viable mutants lacking nicks were also isolated [38]. It was demonstrated for phikF77 [15] that nicks are repaired at early stages of phage infection and reappear at the late stages. It is known that the left end of the T5 is the first to be packed [32]. Mutants lacking one nick were isolated for phikF77 and a phage with such mutation was found in the environment [15]. It is likely that in all phages studied (Fig. 5) the half of the genome with the highest density of nicking is the first to be packed into the phage particle. Based on these data it could be suggested that site specific nicking may facilitate initial stages of phage DNA packaging by increasing flexibility of the molecule. To the best of our knowledge no studies aimed to assess the global distribution of this feature in phage genomes were conducted. It is likely that many more phages with similar DNA organization exist in nature.

Similar to LUZ24 as well as to a number of other phages tf has a genome with short exact direct repeat ends. However, determination of the precise sequences at the termini revealed a unique structure with the blunt right and the 4 nucleotide protruding 3′-left end. There are no similar structures reported for bacteriophage DNA.

**Figure 7:**
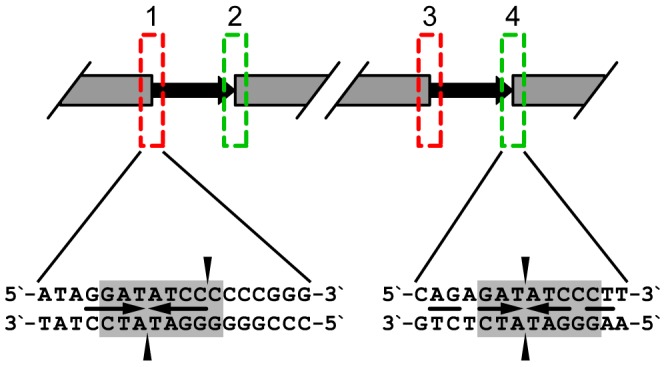
Putative processing of the tf DNA from concatemer substrate. Regions on the concatemer containing terminase recognition sequences are boxed with a red box corresponding designating sequences found at the left end of the genome and a green one – at the right. A putative core recognition site for the terminase enzyme is shaded with grey and positions of cuts are indicated by vertical arrows. Palindrome sequences identified within these sites are shown by horizontal arrowed lines.

The organization of phage DNA ends is thought to reflect its DNA packaging strategy [30]. Therefore, it is likely that tf packages its DNA similar to other phages with short exact direct repeat ends using concatemer substrates as shown in Fig. 7 where a putative core site for terminase recognition in the course of processing is presented by 5′-GATATCcc-3′ sequence. However, considering that two different ends are produced in the course of this sequence cleavage, some details of the phage DNA processing may need to be re-addressed using the tf data.

In summary: The genome of the *P. putida* bacteriophage tf combines two striking features: fourteen localized single-chain interruptions and unique termini, with a blunt right end and a 4 nucleotide 3′-protruding left end. The feature of the genome nicking seems to be wider spread in nature than previously thought and may have a fundamental significance for bacteriophages.

## Supporting Information

Figure S1 **SDS-PAGE analysis of the phage particle proteins.** CsCl gradient purified tf preparation was used. Putative gene products (gp) shown were identified on the basis of molecular weight estimations.
(TIF)

Figure S2 **Identification of localized single-chain interruptions in the bacteriophage tf.** All sequencing reactions were performed on native DNA preparations using phage specific primers complementary to the top strand. (A) Positions of the localized nicks (1 to 14) are manifested by drops of signal (indicated on chromatograms) resulting from stoppages of a DNA polymerase during the read-through a corresponding site. (B) Logo of the sequences flanking sci sites in the tf genome Constructed using http://www.biogenio.com/logo/logo.cgi.
(TIF)

Table S1 **Main features of the bacteriophage tf genome.** ^a)^ Abbreviations used: LTR and RTR, left and right terminal repeat, respectively; tf.N, phage tf open reading frame number N; P, promoter; T, terminator of transcription; sci, single-chain interruption. ^b)^ Only homologues with the best scores are presented. Their characteristics are as fallows: organism/protein name/data base accession number/E-value. References to phage LUZ24, PaP3 and phiMR299-2 proteins can be found in their complete genome GenBank entries (accession numbers AM910650, AY078382 and JN254801, respectively). ^c)^ U, unique, present only in tf genome; GS, group-specific, present also in the genome of at least one additional representative of LUZ24-like phage group (LUZ24, PaP3 and phiMR299-2); CP, conserved in genomes of the phages belonging to the other groups; CB, conserved in bacterial genomes.
(DOCX)
