# Supplementary figures and images for: Genomic Analysis of Pseudomonas putida Phage tf with Localized Single-Strand DNA Interruptions
Source: PLoS One. 2012 Dec 7;7(12):e51163. doi: 10.1371/journal.pone.0051163 (PMC3517423; doi:10.1371/journal.pone.0051163)

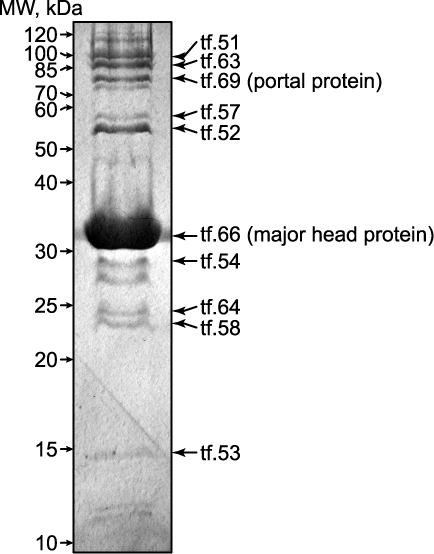

Supplement: Figure S1 — SDS-PAGE analysis of the phage particle proteins. CsCl gradient purified tf preparation was used. Putative gene products (gp) shown were identified on the basis of molecular weight estimations. (TIF) [file pone.0051163.s001.tif]

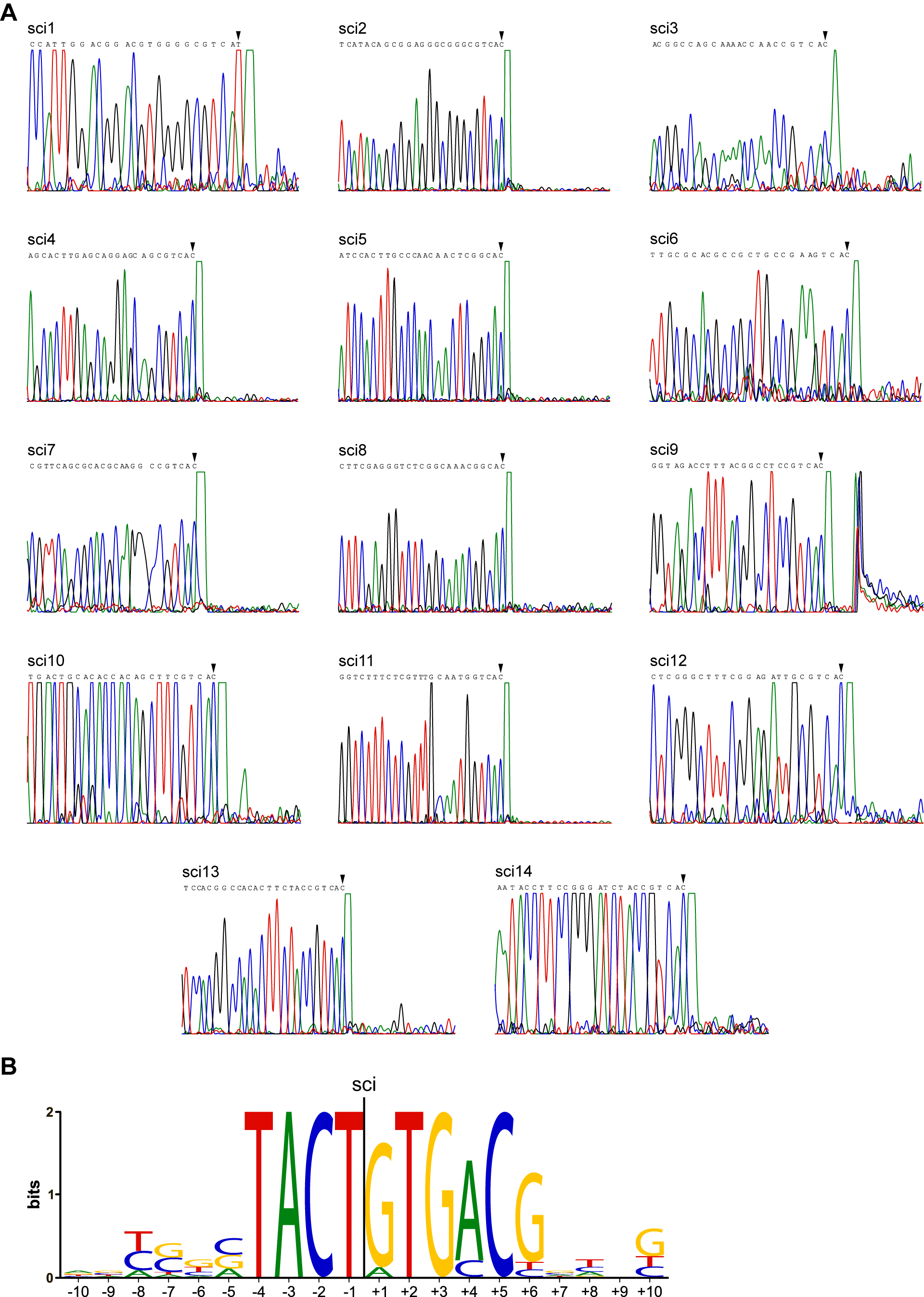

Supplement: Figure S2 — Identification of localized single-chain interruptions in the bacteriophage tf. All sequencing reactions were performed on native DNA preparations using phage specific primers complementary to the top strand. (A) Positions of the localized nicks (1 to 14) are manifested by drops of signal (indicated on chromatograms) resulting from stoppages of a DNA polymerase during the read-through a corresponding site. (B) Logo of the sequences flanking sci sites in the tf genome Constructed using http://www.biogenio.com/logo/logo.cgi. (TIF) [file pone.0051163.s002.tif]
