# Supplementary material for: Genomic Analysis of Pseudomonas putida Phage tf with Localized Single-Strand DNA Interruptions
Source: PLoS One. 2012 Dec 7;7(12):e51163. doi: 10.1371/journal.pone.0051163 (PMC3517423; doi:10.1371/journal.pone.0051163)
Supplement: Table S1 — Main features of the bacteriophage tf genome. a) Abbreviations used: LTR and RTR, left and right terminal repeat, respectively; tf.N, phage tf open reading frame number N; P, promoter; T, terminator of transcription; sci, single-chain interruption. b) Only homologues with the best scores are presented. Their characteristics are as fallows: organism/protein name/data base accession number/E-value. References to phage LUZ24, PaP3 and phiMR299-2 proteins can be found in their complete genome GenBank entries (accession numbers AM910650, AY078382 and JN254801, respectively). c) U, unique, present only in tf genome; GS, group-specific, present also in the genome of at least one additional representative of LUZ24-like phage group (LUZ24, PaP3 and phiMR299-2); CP, conserved in genomes of the phages belonging to the other groups; CB, conserved in bacterial genomes. (DOCX) [file pone.0051163.s003.docx]

**Table S1. Main features of the bacteriophage tf genome**

| Feature^a^ | Strand | Start | Stop | Length  (bp/aa) | G+C  (%) | *M*_r_/pI | Homolog^b^ | Domain (motif) | Predicted function | Gene  Type^c^ |
| --- | --- | --- | --- | --- | --- | --- | --- | --- | --- | --- |
| 3’-end (TAGG) | - | -4 | -1 | 4 |  |  |  |  |  |  |
| 5’-end | + | 1 |  |  |  |  |  |  |  |  |
| LTR | + | 1 | 186 | 186 | 53.8 |  |  |  |  |  |
| P | + | 338 | 412 | 75 | 61.3 |  |  |  |  |  |
| P | + | 485 | 559 | 75 | 54.7 |  |  |  |  |  |
| P | + | 669 | 743 | 75 | 53.3 |  |  |  |  |  |
| P | + | 747 | 821 | 75 | 50.7 |  |  |  |  |  |
| tf.01 | + | 1130 | 1489 | 360/  119 | 50.0 | 13,149/  10.26 |  |  |  | U |
| tf.02 | + | 1501 | 1980 | 480/  159 | 56.9 | 17,667/  9.46 | phage PaP3/ p70/ 8e-35 |  |  | GS, CP |
| tf.03 | + | 1977 | 2087 | 111/  36 | 55.9 | 3,889/  9.70 |  | contains one transmembrane region |  | U |
| sci1 | + | 2102 | 2103 |  |  |  |  |  |  |  |
| tf.04 | + | 2103 | 2396 | 294/  97 | 54.8 | 11,324/  10.58 |  |  |  | U |
| tf.05 | + | 2396 | 2506 | 111/  36 | 52.3 | 3,906/  4.13 |  | contains one transmembrane region |  | U |
| tf.06 | + | 2506 | 2694 | 189/  62 | 50.8 | 6,457/  9.45 |  |  |  | U |
| tf.07 | + | 2938 | 3171 | 234/  77 | 53.0 | 8,515/  5.85 |  |  |  | U |
| tf.08 | + | 3175 | 3411 | 237/  78 | 53.2 | 8,751/  6.92 |  |  |  | U |
| tf.09 | + | 3415 | 3498 | 84/  27 | 58.3 | 3,179/  12.11 |  |  |  | U |
| sci2 | + | 3494 | 3495 |  |  |  |  |  |  |  |
| tf.10 | + | 3726 | 4001 | 276/  91 | 55.4 | 10,476/  9.55 | *Shigella dysentaria* 225-7/ L-asparaginase 2 domain protein/ EIQ57312/ 8e-06 | cd08964; Type II (periplasmic) bacterial L-asparaginase |  | U |
| tf.11 | + | 3982 | 4176 | 195/  64 | 53.8 | 7,504/  10.59 |  |  |  | U |
| tf.12 | + | 4177 | 4377 | 201/  66 | 51.7 | 7,551/  9.58 |  |  |  | U |
| sci3 | + | 4215 | 4216 |  |  |  |  |  |  |  |
| tf.13 | + | 4390 | 4767 | 378/  125 | 57.4 | 14,173/  8.97 | phage phiMR299-2/ 17C_055c/ 4e-04 |  |  | GS |
| P | + | 4763 | 4832 | 75 | 42.9 |  |  |  |  |  |
| tf.14 | + | 5017 | 5271 | 255/  84 | 57.6 | 9,328/  8.90 |  |  |  | U |
| sci4 | + | 5267 | 5268 |  |  |  |  |  |  |  |
| tf.15 | + | 5268 | 5483 | 216/  71 | 58.8 | 8,147/  8.76 |  | contains two transmembrane regions |  | U |
| tf.16 | + | 5493 | 5714 | 222/  73 | 53.2 | 8,341/  6.08 |  | cd03690; Tet_II: This subfamily represents domain II of ribosomal protection proteins TetM and TetO |  | U |
| tf.17 | + | 5776 | 6669 | 894/  297 | 55.0 | 33,260/  8.33 |  |  |  | U |
| sci5 | + | 6625 | 6626 |  |  |  |  |  |  |  |
| tf.18 | + | 6666 | 7589 | 924/  307 | 56.3 | 33,725/  8.26 | phage LUZ24/ gp15/ 1e-04 |  |  | GS |
| tf.19 | + | 7593 | 8381 | 789/  262 | 59.1 | 28,926/  4.99 | phage PaP3/ p48/ 1e-100;  phage phiMR299-2/ 17C_049c/ 1e-100 | IPRO25681; Phage phiEco32-like COOH-NH2 ligase – type 2 |  | GS, CP, CB |
| tf.20 | + | 8378 | 8959 | 582/  193 | 56.4 | 21,572/  8.36 | phage LUZ24/ gp17/ 1e-48 |  |  | GS |
| sci6 | + | 8633 | 8634 |  |  |  |  |  |  |  |
| tf.21 | + | 8935 | 10116 | 1182/  393 | 58.8 | 44,093/  5.66 | phage phiMR299-2/ 17C_047c/ 1e-83;  phage LUZ24/ gp18/ 1e-83 | Pfam 12224; Amidoligase_2 | amidoligase | GS, CB |
| sci7 | + | 9969 | 9970 |  |  |  |  |  |  |  |
| tf.22 | + | 10145 | 11740 | 1596/  531 | 56.7 | 58,312/  6.93 | phage LUZ24/ gp19/ 3e-132 | IPRO17932; Glutamine amido-transferase type 2 domain | glutamine amidotransferase | GS, CB |
| tf.23 | + | 11740 | 11949 | 210/  69 | 54.3 | 8,067/  5.19 | phage phiMR299-2/ 17C_045c/ 2e-14;  phage LUZ24/ gp20/ 2e-14 |  |  | GS |
| tf.24 | + | 12275 | 13147 | 873/  290 | 58.0 | 31,734/  7.89 | phage PaP3/ p43/ 2e-103 | IPRO13816; ATP-grasp fold subdomain 2 |  | GS, CB |
| sci8 | + | 12544 | 12545 |  |  |  |  |  |  |  |
| tf.25 | + | 13144 | 13587 | 444/  147 | 55.4 | 16,662/  4.95 | phage LUZ24/ gp22/ 9e-38 | IPRO9288; AIG2-like;  IPRO13024; Butirosin biosynthesis, BtrG-like |  | GS, CB |
| tf.26 | + | 13584 | 13955 | 372/  123 | 56.5 | 13,466/  5.35 | phage LUZ24/ gp23/ 8e-25 | PRK09562; mazG, nucleoside tri-phosphate pyro-phosphohydrolase |  | GS |
| sci9 | + | 13598 | 13599 |  |  |  |  |  |  |  |
| tf.27 | + | 13952 | 15655 | 1704/  567 | 54.5 | 63,711/  5.97 | phage PaP3/ p40/ 0.0;  phage LUZ24/ gp24/ 0.0;  phage phiMR299-2/ 17C_041c/ 0.0 | IPRO07694; DNA helicase, DNAB-like, C-terminal;  SSF56731, DNA primase core | primase/helicase | GS, CP, CB |
| tf.28 | + | 15630 | 16136 | 507/  168 | 51.7 | 19,391/  5.02 | phage PaP3/ p39/ 2e-70 | cd06127; DEDDh 3’-5’ exonuclease domain family | 3’-5’ exonuclease | GS |
| sci10 | + | 15698 | 15699 |  |  |  |  |  |  |  |
| tf.29 | + | 16115 | 16429 | 315/  104 | 53.0 | 11,483/  10.65 | phage phiMR299-2/ 17C_040c/ 9e-08 | contains one transmembrane region |  | GS |
| tf.30 | + | 16445 | 16591 | 147/  48 | 53.1 | 5,666/  11.38 |  |  |  | U |
| tf.31 | + | 16588 | 16752 | 165/  54 | 53.3 | 5,986/  8.80 |  |  |  | U |
| tf.32 | + | 16746 | 17042 | 297/  98 | 49.5 | 11,205/  9.89 | phage LUZ24/ gp31/ 5e-15 | contains two transmembrane regions | holin | GS |
| P | + | 17007 | 17081 | 75 | 42.7 |  |  |  |  |  |
| tf.33 | + | 17101 | 17223 | 123/  40 | 49.6 | 4,200/  9.70 |  | contains one transmembrane region |  | U |
| tf.34 | + | 17220 | 17426 | 207/  68 | 59.4 | 7,567/  3.66 |  |  |  | U |
| tf.35 | + | 17426 | 17662 | 237/  78 | 56.5 | 9,047/  10.34 |  |  |  | U |
| tf.36 | + | 17664 | 17801 | 138/  45 | 49.3 | 5,232/  6.93 |  |  |  | U |
| tf.37 | + | 17801 | 18208 | 408/  135 | 57.1 | 15,313/  4.65 |  |  |  | U |
| tf.38 | + | 18218 | 19858 | 1641/  546 | 52.8 | 60,856/  9.49 | phage PaP3/ p32/ 0.0;  phage phiMR299-2/ 17C_033c/ 0.0;  phage LUZ24/ gp34/ 0.0 | IPRO01098; DNA-directed DNA poly-merase, family A, palm domain;  SSF56672; DNA/RNA polymerases | DNA polymerase | GS |
| tf.39 | + | 19868 | 20059 | 192/  63 | 49.0 | 7,692/  9.92 | phage phiR1-RT/ gp_39/ YP_006382499/  4e-16 |  |  | CP |
| P | + | 20036 | 20110 | 75 | 36.0 |  |  |  |  |  |
| tf.40 | + | 20115 | 20726 | 612/  203 | 53.1 | 22,240/  4.75 | phage PaP3/ p31/ 2e-47 | IPRO12340; Nucleic acid-binding, OB-fold |  | GS |
| sci11 | + | 20794 | 20795 |  |  |  |  |  |  |  |
| tf.41 | + | 20795 | 21139 | 345/  114 | 53.0 | 12,408/  7.09 | phage phiMR299-2/ 17C_031c/ 1e-39;  phage PaP3/ p30/ 1e-39 |  |  | GS, CP |
| tf.42 | + | 21235 | 21618 | 384/  127 | 53.1 | 13,698/  5.50 | phage phiMR299-2/ 17C_030c/ 3e-22;  phage PaP3/ p29/ 3e-22 |  |  | GS |
| tf.43 | + | 21494 | 21730 | 237/  78 | 49.8 | 9,101/  9.65 |  |  |  | U |
| tf.44 | + | 21727 | 22650 | 924/  307 | 51.6 | 34,963/  5.07 | phage PaP3/ p28/ 2e-132 | IPRO20045; 5’-3’ exonuclease, C-terminal domain;  IPRO08918; Helix-hairpin-helix motif;  CATH 3.40.50.1010; 5’-nuclease | 5’-3’ exonuclease | GS |
| tf.45 | + | 22583 | 23506 | 924/  307 | 54.0 | 35,668/  5.05 | phage PaP3/ p27/ 8e-32 | CATH 3.30.460.10; Beta polymerase, domain 2;  cd05398; Nucleotidyl-transferase domain of class II CCA-adding enzymes |  | GS, CB |
| tf.46 | + | 23370 | 23801 | 432/  143 | 52.1 | 17,050/  5.72 | phage 7-11/ SaPh711_gp029/ 1e-23 | IPRO08029; Bacteriophage T7, GP3, endonuclease I;  IPRO11578; Restriction endo-nuclease, FokI, C-terminal | endonuclease | GS, CP |
| tf.47 | + | 23785 | 24546 | 762/  253 | 51.6 | 28,836/  7.97 | phage LUZ24/ gp44/ 5e-136 |  |  | GS, CP |
| tf.48 | + | 24598 | 24825 | 228/  75 | 52.6 | 8,637/  8.87 |  |  |  | U |
| sci12 | + | 24720 | 24721 |  |  |  |  |  |  |  |
| tf.49 | + | 24806 | 25027 | 222/  73 | 53.2 | 8,561/  5.11 |  |  |  | U |
| T | + | 25098 | 25133 | 36 | 55.6 |  |  |  |  |  |
| T | - | 25126 | 25092 | 35 | 57.1 |  |  |  |  |  |
| tf.50 | - | 25395 | 25132 | 264/  87 | 50.0 | 8,811/  6.29 |  |  |  | U |
| tf.51 | - | 28460 | 25395 | 3066/  1021 | 54.3 | 108,592/  5.81 | phage LUZ24/ gp50/ 0.0;  phage phiMR299-2/ 17C_019/ 0.0;  phage PaP3/ p19/ 0.0 | Pfam 04306; Protein of unknown function (DUF456) | phage particle protein | GS |
| tf.52 | - | 29925 | 28462 | 1464/  487 | 54.7 | 51,703/  7.09 | phage PaP3/ p18/ 3e-33 |  | phage particle protein | GS |
| tf.53 | - | 30315 | 29935 | 381/  126 | 52.8 | 13,251/  6.31 |  |  |  | U |
| tf.54 | - | 31250 | 30312 | 939/  312 | 53.2 | 32,209/  4.70 | phage LUZ24/ gp53/ 3e-90 |  | phage particle protein | GS |
| tf.55 | - | 31665 | 31231 | 435/  144 | 44.8 | 16,344/  5.35 | phage LUZ24/ gp54/ 2e-43 |  |  | GS |
| P | - | 31744 | 31670 | 75 | 29.3 |  |  |  |  |  |
| tf.56 | - | 32028 | 31747 | 282/  93 | 54.6 | 10,185/  4.64 | phage LUZ24/ gp55/ 1e-11 |  |  | GS |
| tf.57 | - | 33587 | 32028 | 1560/  519 | 52.7 | 57,232/  5.55 | phage LUZ24/ gp56/ 0.0;  phage PaP3/ p13/ 0.0  phage phiMR299-2/ 17C_013/ 0.0 |  | phage particle protein | GS, CP, CB |
| tf.58 | - | 34244 | 33591 | 654/  217 | 54.1 | 22,592/  6.35 | phage PaP3/ p12/ 9e-31 | SSF88874; Receptor binding domain of short tail fiber protein gp12 superfamily | tail fiber protein | GS, CP, CB |
| sci13 | + | 33849 | 33850 |  |  |  |  |  |  |  |
| tf.59 | - | 34440 | 34234 | 207/  68 | 52.7 | 7,289/  5.09 |  |  |  | U |
| tf.60 | - | 34739 | 34452 | 288/  95 | 52.8 | 10,248/  8.11 | phage tf/ tf.61/  6e-05 |  |  | U |
| tf.61 | - | 34999 | 34736 | 264/  87 | 50.8 | 9,079/  7.01 | phage tf/ tf.60/  6e-05 |  |  | U |
| tf.62 | - | 35177 | 35010 | 168/  55 | 51.8 | 6,213/  8.34 |  |  |  | U |
| tf.63 | - | 37837 | 35186 | 2652/  883 | 51.1 | 95,812/  5.26 | *Burkholderia ambifaria* Mex-5/ BamMEX5DRAFT_2260/ ZP_02906906/  3e-72 | IPRO13830; Esterase, SGNH hydrolase-type;  Pfam 12708; Pectate lyase superfamily protein | phage particle protein associated with glycosidase activity | CB |
| sci14 | + | 37115 | 37116 |  |  |  |  |  |  |  |
| tf.64 | - | 38517 | 37837 | 681/  226 | 52.3 | 26,478/  5.34 | *Candidatus Puniceispirillum marinum* IMCC1322/ SAR116_0371/  2e-16 |  | phage particle protein | GS, CP, CB |
| tf.65 | - | 38785 | 38453 | 333/  110 | 49.8 | 12,643/  6.14 | phage PaP3/ p08/ 3e-13 |  |  | GS |
| T | - | 38830 | 38804 | 27 | 66.7 |  |  |  |  |  |
| tf.66 | - | 39797 | 38841 | 957/  318 | 54.2 | 34,847/  8.63 | phage LUZ24/ gp62/ 2e-128 |  | major head protein | GS, CP, CB |
| tf.67 | - | 40814 | 39810 | 1005/  334 | 50.2 | 37,751/  4.34 | phage LUZ24/ gp63/ 1e-54 |  | scaffolding protein | GS, CB |
| tf.68 | - | 41053 | 40814 | 240/  79 | 51.7 | 9,294/  4.84 | phage LUZ24/ gp64/ 5e-06 |  |  | GS |
| tf.69 | - | 43173 | 41053 | 2121/  706 | 50.6 | 80,469/  4.99 | phage phiMR299-2/ 17C_004/ 0.0;  phage PaP3/ p04/ 0.0;  phage LUZ24/ gp65/ 0.0 |  | portal protein | GS, CP, CB |
| tf.70 | - | 44612 | 43173 | 1440/  479 | 51.9 | 53,636/  6.78 | phage phiMR299-2/ 17C_003/ 0.0;  phage PaP3/ p03/ 0.0;  phage LUZ24/ gp66/ 0.0 | IPRO04921; Terminase, large subunit | terminase, large subunit | GS, CP, CB |
| tf.71 | - | 45112 | 44612 | 501/  166 | 50.1 | 18,535/  9.25 | phage PaP3/ p02/ 1e-69 | IPRO02196; Glycoside hydrolase, family 24 | lysozyme | GS, CP, CB |
| tf.72 | - | 45503 | 45045 | 459/  152 | 48.4 | 16,718/  5.97 | phage phiMR299-2/ 17C_001/ 8e-73 | COG3728; Phage terminase, small subunit | terminase, small subunit | GS, CP, CB |
| P | - | 45595 | 45521 | 75 | 25.3 |  |  |  |  |  |
| P | - | 45857 | 45783 | 75 | 33.3 |  |  |  |  |  |
| RTR | + | 46082 | 46267 | 186 | 53.8 |  |  |  |  |  |
| 3’-end | + | 46267 |  |  |  |  |  |  |  |  |
| 5’-end | - | 46267 |  |  |  |  |  |  |  |  |
